# Recent Advances in Prehospital and In-Hospital Management of Patients with Severe Trauma

**DOI:** 10.3390/jcm14072208

**Published:** 2025-03-24

**Authors:** Jung-Youn Kim, Oh Hyun Kim

**Affiliations:** 1 Department of Emergency Medicine, College of Medicine, Korea University, Seoul 08308, Republic of Korea; 2 Department of Emergency Medicine, Yonsei University Wonju College of Medicine, Wonju 26426, Republic of Korea

**Keywords:** injuries, hemorrhage, emergency medical services, advanced trauma life support care

## Abstract

**Background:** Trauma is a major global public health concern. Many countries are working to reduce preventable deaths; however, the mortality rate remains higher than their goal, indicating a need for continuous development in trauma care, including further improvements across the system. This article explores recent developments and updated guidelines for both prehospital emergency care and in-hospital trauma management, emphasizing evidence-based and patient-centered approaches. **Current concepts:** In the prehospital phase, the primary focus is on early and aggressive hemorrhage control using techniques such as tourniquet application, wound packing, and permissive hypotension as standard practices. Advancements in this field, including intraosseous vascular access and tranexamic acid administration, have improved patient outcomes. The emphasis on structured assessments, particularly “circulation, airway, breathing” (CAB) assessments, underscores the importance of managing life-threatening hemorrhages. During the in-hospital phase, the primary focus is on controlling bleeding. Protocols emphasize the judicious administration of fluids to prevent over-resuscitation and mitigate the risk of exacerbating coagulopathy. Efficient transfusion strategies are implemented to address hypovolemia, while ensuring balanced ratios of blood products. Furthermore, the implementation of advanced interfacility transfer systems and communication tools such as “Situation, Background, Assessment, Recommendation” (SBAR) plays a pivotal role in optimizing patient care and reducing delays in definitive treatment. **Discussion and Conclusions:** This review highlights the importance of implementing advanced strategies to align with international standards and further decrease the rate of preventable trauma-related deaths. Strengthening education and optimizing resource allocation for both prehospital and hospital-based trauma care are essential steps toward achieving these objectives.

## 1. Introduction

Trauma is a major global public health concern. It accounts for a considerable percentage of deaths, particularly among young adults, and its impact is disproportionately higher in low- and middle-income countries where resources for trauma care are often limited. Trauma-related injuries include road traffic accidents, falls, violence, and workplace injuries, all of which can result in a wide spectrum of outcomes, from minor injuries to life-threatening conditions [1,2,3,4,5]. While many countries have made substantial progress in reducing preventable trauma deaths, the mortality rate remains higher than desired. This suggests that despite improvements, there is still a critical need for continuous interest to innovation and systemic improvements in trauma care [6]. Recent advancements in trauma care have focused on the integration of evidence-based protocols into both prehospital and in-hospital settings. These innovations are aimed at improving patient outcomes through timely, appropriate, and coordinated interventions across the continuum of care. Specifically, prehospital emergency medical services (EMSs), which include rapid trauma assessment and initial stabilization, play a crucial role in determining the prognosis of trauma patients. Specialized in-hospital trauma care, ranging from surgical interventions to intensive care, is vital for managing complex trauma cases. The development of trauma care guidelines, such as advanced trauma life support (ATLS) and the European Trauma Guidelines, reflect the evolving landscape of trauma management. These guidelines, alongside technological advancements in diagnostics and treatment strategies, have reshaped the approach to trauma care [7]. In particular, revised guidelines and treatment protocols introduced in recent years emphasize a more integrated approach to trauma care, highlighting the importance of early intervention, multidisciplinary collaboration, and the optimization of care pathways. As trauma care continues to evolve, it is essential to remain up to date with these changes to enhance patient survival rates and recovery outcomes.

This article explores the recent developments and updated guidelines for both prehospital emergency care and in-hospital trauma management, emphasizing evidence-based and patient-centered approaches. In particular, efficient prehospital first aid and specialized trauma care are key components of managing patients with trauma. Various revised guidelines and new treatment strategies proposed in recent years suggest that the current trauma management paradigm is changing. In this article, we review the notable changes in prehospital first aid and specialized trauma care based on recent national and international literature and revised guidelines.

## 2. Prehospital and Initial In-Hospital Care of Patients with Severe Trauma

### 2.1. Prehospital Care of Patients with Severe Trauma

Critical trauma assessment for prehospital paramedics consists of (1) securing and protecting the airway; (2) assessing respiratory function; (3) assessing circulation and bleeding control, accessing vascularization, and adequate fluid resuscitation; and (4) screening for neurologic impairment and identifying life-threatening hemorrhage by skin exposure [8]. While the traditional ABC approach (airway, breathing, circulation) has been utilized for decades, a recent shift to the CAB (circulation, airway, breathing) approach, which first checks circulation and focuses on initial bleeding control, has been noted [9].

#### 2.1.1. Airway Management

Airway assessment and management are essential when evaluating patients with trauma. It is important to focus on basic airway management, including airway positioning, the use of nasopharyngeal and oropharyngeal airway devices, and ventilation using a bag-valve mask. These initial interventions can facilitate rapid and safe transportation to hospitals. However, complex injuries such as altered consciousness, airway compromise, oropharyngeal injury, or airway obstruction may require advanced airway management [10,11]. Intubation in a prehospital setting is performed by experienced paramedics or field emergency medical technicians with sufficient experience. The primary goal of ventilation in the trauma patient is to maintain airway patency and adequate oxygenation. Intubation is recommended without delay in cases of airway obstruction, altered consciousness (GCS ≤ 8), hypoventilation, or hypoxemia [12]. It is essential to avoid hypoxemia and hypoxemia, except in situations such as imminent exsanguination. In trauma patients, normoventilation is recommended, with hyperventilation only suggested as a life-saving measure in cases of cerebral herniation. The use of endotracheal intubation, although essential in certain conditions, can be risky and requires skill. Supraglottic airway devices have been explored as an alternative, but recent studies show no superiority over endotracheal intubation [13]. Hypoxemia, particularly in patients with traumatic brain injury (TBI), should initially be treated with high levels of oxygen, but prolonged hyperoxia may increase mortality, particularly in TBI patients. Hyperventilation should generally be avoided as it can lead to adverse effects such as reduced cerebral blood flow and hypotension, but it may temporarily reduce intracranial pressure in cases of imminent brain herniation [14,15,16,17].

#### 2.1.2. Chest Decompression

Tension pneumothorax is a life-threatening complication of chest injuries that requires prompt decompression therapy. This condition is suspected upon the presence of unilateral lung sound loss, dyspnea, hypoxia, and hemodynamic instability. In some countries, rapid on-site needle decompression or finger tube placement is recommended for suspected tension [18,19]. However, in other countries, this is not possible because needle decompression is not currently included in the scope of paramedics. Therefore, if tension pneumothorax is suspected, the best response is to quickly transport the patient to a medical facility for immediate chest decompression. Some guidelines recommend prehospital ultrasonography (PHUS) to detect pneumothorax, hemothorax, and/or free abdominal fluid in the prehospital phase, but in some countries, this is not legally within the scope of practice of paramedics [7].

#### 2.1.3. Intravenous (IV) and Intraosseous (IO) Injection Access

Rapid vascularization is essential for the timely resuscitation of patients with trauma. For this purpose, obtaining at least two large-bore intravenous lines of 16 gauge or larger is generally recommended. If a peripheral vein is not available, vascular access is often obtained via intraosseous injection, which is faster and safer than peripheral venous access [20]. If a pressure bag is used, a 15-gauge needle can infuse fluids at the same rate as an 18-gauge needle [21]. Intraosseous injection access is not included in the scope of paramedic practice in many countries; therefore, there are limited alternatives to intravenous access. The choice between IV and IO is up to the paramedic within the legal framework and practical guidelines of each country.

#### 2.1.4. Prehospital Traumatic Hemorrhage Management Strategies

Recently, prehospital trauma care concepts have focused on minimizing preventable deaths due to hemorrhagic shock by prioritizing the management of massive hemorrhages before ABC access (X-ABC). This is performed to quickly control situations that can lead to bleeding and death in the absence of immediate intervention in the field, such as extremity bleeding, and to prevent hypovolemic shock and organ damage. In the subsequent period, fluid resuscitation in the circulatory phase is a critical intervention in the management of patients with traumatic hemorrhage. Until recently, Ringer’s lactate or normal saline was used; however, recent studies have suggested that the administration of large volumes of fluids may not be beneficial and may even be harmful [22,23]. Therefore, a strategy of minimizing fluid administration and maintain permissive hypotension until arrival at a medical facility is recommended except the patient with TBI and/or spinal injury [24]. Permissive hypotension should be carefully considered and may be contraindicated in elderly patients, those with chronic arterial hypertension, and those with TBI or spinal cord injury. Adequate perfusion pressure is important in these patients [25].

There are no clear guidelines for the use of blood products in the prehospital setting. Some studies suggest positive outcomes with plasma and packed red blood cells, but consistent evidence is still limited [7,26,27,28,29,30].

Bleeding is the leading cause of preventable trauma-related death. Although direct pressure compression is recommended as the first step in controlling bleeding, it may not be sufficient if the injury is extensive or in situations of vascular compromise. Commercially available tourniquets can be used to quickly control extremity bleeding, and these measures have been shown to significantly improve patient survival [31]. Tourniquets are safe, especially when the application time is <2 h [32]. For areas where tourniquets are difficult to apply (perineum, armpit, neck, etc.), wound packing is effective and gauze with a hemostatic agent is ideal; however, plain sterile gauze also works equally fine. Successful perineal hemorrhage control with transitional tourniquets has been reported in military settings; however, more evidence is needed to prove their effectiveness in civilian prehospital settings [33]. Meanwhile, Resuscitative Endovascular Balloon Occlusion of the Aorta (REBOA) is a method of controlling bleeding by temporarily blocking arterial blood flow [34]. Most REBOA studies have been conducted in trauma centers; however, some case reports have outlined prehospital applications. When transferring patients with bleeding trauma by well-equipped air transport or ambulance, the medical staff may consider using REBOA. However, there is very limited experience [35].

Several studies have reported that tranexamic acid administration is associated with improved survival, relative reduction in all-cause mortality, and reduction in bleeding-related complications, including hypoperfusion, organ failure, and transfusion requirements [36]. In addition, the hypercoagulable states in the tranexamic acid group were similar to those in the non-tranexamic acid group [37]. For the effective use of tranexamic acid, it is important to begin administration within 1 h of the onset of bleeding and avoid administration after 3 h of injury. The current recommended protocol is to administer an initial 1 g bolus within 10 min, followed by an additional 1 g bolus over the next 8 h. Intraosseous or intramuscular injections can be administered if intravenous access is difficult to achieve [38].

#### 2.1.5. Spinal Immobilization or Spinal Motion Restriction

If a cervical spine injury is suspected, paramedics should apply a cervical spine brace according to the prehospital trauma care guidelines. At this point, paramedics can use validated tools such as the National Emergency X-Radiography Utilization Study (NEXUS) to identify patients who do not require a cervical spine brace. While there is little evidence that the use of cervical spine braces reduces morbidity or mortality, harmful effects have been reported, including worsening of intubation conditions and increased intracranial pressure [39,40]. In addition, there is a lack of evidence that spinal immobilization plates contribute to improved neurologic outcomes despite their strong association with pressure ulcer development and ventilation restriction. Therefore, it is inappropriate to apply them uniformly to all patients with trauma. Spinal motion restriction is not recommended for patients with penetrating trauma because it is associated with increased mortality [41].

#### 2.1.6. Pelvic Binder

Pelvic fractures can cause significant bleeding in patients with trauma. A pelvic binder can be used in the prehospital setting to reduce bleeding before the final treatment of a pelvic fracture. Paramedics should strongly suspect pelvic ring fractures in patients with multiple traumas or when pelvic injury is suspected and hemodynamic instability is present. In these situations, early empirical application of pelvic binders is recommended. Although the prehospital use of pelvic binders is controversial, prehospital trauma care and specialized trauma care guidelines recommend their application in unstable patients with suspected pelvic fractures [42,43,44] because of the relatively low cost, low risk of complications, and potential benefits.

#### 2.1.7. Treatment of Traumatic Brain Injury

Traumatic brain injury is the leading cause of death in patients with trauma [45]. In the prehospital phase, secondary causes of brain injury, such as hypotension, hypoxemia, and hypothermia, should be aggressively corrected, as each of these is independently associated with increased mortality [46]. End-expiratory carbon dioxide should be maintained in the normal range (35–40 mmHg) and prophylactic hyperventilation should be avoided, as it may worsen the neurologic prognosis [47]. In cases of true brain herniation, hyperventilation can be used to temporarily reduce end-expiratory carbon dioxide to 28–35 mmHg; however, this is only a temporary measure until further treatment. To reduce intracranial pressure, excessive cervical collar compression should be avoided, the head should be elevated by at least 30°, appropriate sedation or analgesia should be administered, and hyperosmotic therapy should be considered. Ultimately, a neurosurgical intervention may be required. However, the empirical use of hyperosmolar therapy in prehospital settings has not been shown to be beneficial [48].

#### 2.1.8. Selecting a Transport Method

There are various means of transporting patients with trauma to a definitive care facility, and it is recommended that the choice of care facility be guided by national field triage guidelines (e.g., Standardized Guidelines for Emergency Medical Care for First Responders) [49]. Minimizing on-scene time during ambulance transport is critical, and decreased survival rates have been reported with longer on-scene times. Therefore, paramedics should focus on time-sensitive and immediate life-threatening conditions. In contrast, helicopter emergency medical services (HEMSs) can be utilized to provide rapid transport, additional treatment, and a higher level of care when there is a time advantage over ground transport. Although stating that HEMSs provide clear benefits to patients with trauma is controversial, several national studies have reported benefits in patients with severe trauma [50,51,52].

#### 2.1.9. Involvement of Bystanders

Non-medical first responders (e.g., police officers) and bystanders have a critical opportunity to provide life-saving care to patients with trauma before emergency services arrive. Similarly to CPR training, public health interventions to educate civilians on essential trauma care skills such as bleeding control have been successful abroad. The “Stop the Bleed” campaign, led by the American College of Surgeons, is a prime example of this, promoting widespread public education and the availability of bleeding control kits in public places [53]. Similar education or training should be encouraged so that communities can contribute to the response of patients with trauma.

#### 2.1.10. Traumatic Cardiac Arrest (TCA)

Traumatic cardiac arrest (TCA) has a high mortality rate, but recent studies have shown that survival with good to moderate neurological recovery is possible. Successful resuscitation of TCA relies on immediate and simultaneous treatment of reversible causes following a systematic approach. It requires rapid teamwork and multiple interventions. The complexity of managing TCA involves rapid assessment and integration of available information and rapid decision making. The HOTT protocol is a systematic approach to treating the most common reversible causes of TCA. The HOTT protocol, which addresses hypovolemia, hypoxia, tension pneumothorax, and cardiac tamponade, is the foundation of TCA management. Advanced treatment techniques, such as cardiopulmonary resuscitation (CPR), resuscitative thoracotomy (RT), and intra-aortic balloon occlusion (REBOA), help improve outcomes. Approaches focus on metabolic disturbances (e.g., hyperkalemia, calcium imbalance) and hemostatic resuscitation are important [54,55]. The use of epinephrine in the treatment of cardiac arrest patients is common, but there is controversy about its clear survival benefit in TCA patients in prehospital and in-hospital settings [56,57]. The use of external cardiac massage devices has advantages and disadvantages in prehospital cardiac arrest caused by trauma [4,58,59,60].

### 2.2. Initial In-Hospital Care of Patients with Severe Trauma

**Figure 1:**
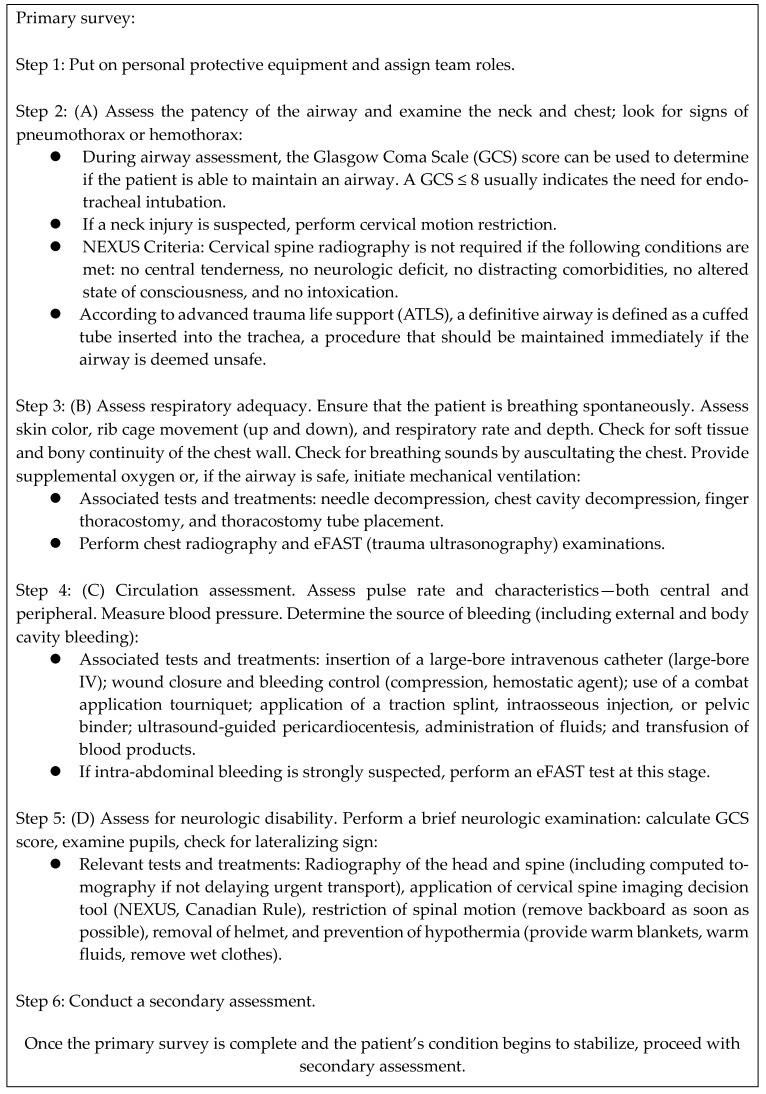
Summary of the primary survey in advanced trauma life support (ATLS). Adapted from the 10th edition of the Advanced Trauma Life Support (ATLS) Student Course Manual, 2018 [61].

**Figure 2:**
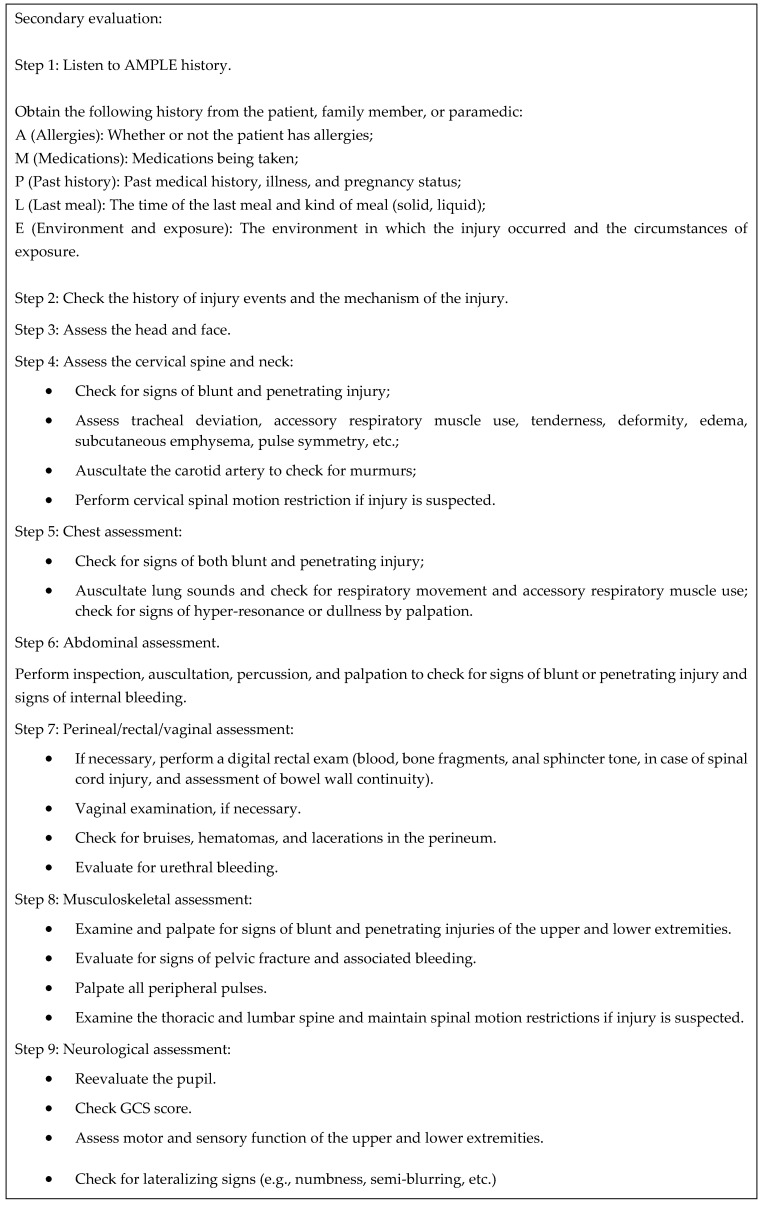
Summary of the secondary survey in advanced trauma life support (ATLS). Adapted from the 10th edition of the Advanced Trauma Life Support (ATLS) Student Course Manual, 2018 [61].

Advanced trauma life support was developed by the American College of Surgeons in 1978; it standardizes the principles of initial assessment, stabilization, and management of trauma patients worldwide. Therefore, it is important for physicians who care for patients with severe trauma to be familiar with this condition. In the initial approach adopted for patients with trauma, a structured and systematic primary survey should be performed first. The primary assessment follows the ABCDE approach, which involves the sequential prioritization of airway, breathing, circulation, neurologic disability, and patient exposure, that is, airway and breathing problems should be addressed before circulation problems are addressed. This can be repeated as the patient’s condition changes. A secondary assessment is performed when the primary assessment is complete and the patient’s condition improves. The secondary assessment includes a full-body assessment from head to toe, and any abnormal findings should be recorded. It also includes hypothermic prophylaxis. Elements of the primary and secondary assessments are shown in Figure 1 and Figure 2.

#### 2.2.1. Airway

The term rapid-sequence intubation has been replaced with drug-assisted intubation. This term emphasizes flexible and safe airway management that allows for patient-optimized drug selection and administration strategies, reflecting the fact that in real clinical situations, approaches vary depending on a number of variables, including the patient’s hemodynamic status, level of consciousness, and mechanism of injury. According to the ATLS 10th Edition, a definitive airway is defined as a cuffed tube positioned within the trachea, which can be achieved by tracheal intubation, cricothyrotomy, or tracheostomy. The usefulness of video laryngoscopy for airway management in patients with severe trauma has been emphasized.

#### 2.2.2. Breathing

Blunt chest injuries occur in >10% of patients with trauma worldwide, with mortality rates varying from 4 to 20% [62]. Immediate life-threatening chest injuries identified in the primary survey include tension pneumothorax, massive hemothorax, and thoracic aortic injury. Traditionally, the insertion of a large-bore chest tube has been recommended for the treatment of hemothorax; however, recent studies have shown that tube size (diameter) has no significant effect on the ability to drain a fresh traumatic hemothorax [63]. Therefore, relatively small chest tubes (28–32 Fr) are recommended for acute hemothorax. Tension pneumothorax can be treated with tube placement or needle decompression in the prehospital setting. The location of needle decompression for pneumothorax has been revised; previously, the midclavicular line in the second intercostal space was recommended. However, a recent systematic review and meta-analysis comparing the midclavicular line to the anterior axillary line site indicated a shorter insertion depth (3.42 cm vs. 4.28 cm) for the anterior axillary line. Therefore, in adults, the needle must be inserted in the fourth or fifth intercostal space at the anterior axillary line [64,65]. For pediatric patients, the needle must be inserted in the second intercostal space at the midclavicular line, as before. Other changes included the inclusion of tracheobronchial tree injury as a major life-threatening chest injury during the primary evaluation.

#### 2.2.3. Circulation

In the four-stage classification of hemorrhagic shock, the categories of pulse pressure and base deficit were added (Table 1). This was one of the most important revisions in advanced trauma life support. Both indicators are highly correlated with the degree of hypovolemia due to bleeding [66,67]. In particular, trends rather than absolute values are important and can help clinicians estimate blood loss and transfusion needs. Up to 1 L of warm fluid is recommended for stage 1 or 2 hemorrhages, and resuscitation with blood products, rather than additional fluids, is recommended for patients with ≥stage 2 hemorrhages [68]. In other words, 1 L of warm crystalloid fluid should be started and rapidly switched to blood products in the cases of unresponsiveness. There is a risk of increased mortality if >1.5 L of crystalloid fluid is administered. Transfusions of >10 units of blood within 24 h or >4 units of blood in 1 h are “massive transfusions”, and early administration of 1 U of concentrated red blood cells, 1 U of fresh frozen plasma, and 1 U of platelets at a 1:1:1 ratio can prevent coagulopathy and thrombocytopenia [69].

Additional revisions to the circulation phase include the use of an ≥18 gauge bore for intravenous access and the use of intraosseous injections when peripheral access is difficult [70]. Tourniquet use is recommended for the treatment of severe extremity bleeding. Management of patients receiving new anticoagulants is necessary. Tranexamic acid and prothrombin concentrates may be used in patients with severe trauma and bleeding [71]. Tranexamic acid can be used within 3 h of the incident, with a bolus of 1 g in the first 10 min and another bolus of 1 g over the next 8 h. However, the 2019 CRASH-3 trial suggests that TXA should be used cautiously in patients with isolated severe TBI (GCS score ≤8 combined with other suggestive clinical findings) and no evidence of associated extracranial injury [72,73].

Viscoelastic methods (thromboelastography, TEG; rotational thromboelastometry, ROTEM) are recommended as useful diagnostic tests in patients with bleeding and can help assess the blood coagulation status and guide transfusion decisions. As indicated in Figure 1, Focused Assessment with Sonography for Trauma (FAST) or e-FAST is an important assessment tool that should not be omitted from the primary survey [74,75]. FAST is useful for identifying thoracic intra-abdominal injuries in patients with severe trauma and can play a crucial role in rapidly determining the need for operation in patients with unstable vital signs [7]. Recent advances in computed tomography (CT) imaging technology have made it possible to perform contrast-enhanced whole-body CT to identify potential sources of bleeding according to hospital protocols [76,77,78]. The proximity of the emergency department to the CT department has been shown to affect patient survival, and some institutions are setting up hybrid rooms where CT scans can be performed directly in the resuscitation room [79,80]. The presence of a well-organized and structured trauma team affects the resuscitation of hemodynamically unstable patients [81].

#### 2.2.4. Neurological Assessment

In advanced trauma life support, the Glasgow Coma Scale (GCS) is useful for assessing traumatic brain injury, and this neurological assessment was revised in 2016 to consider the Brain Trauma Foundation’s guidelines [82,83]. The revised GCS utilizes pressure stimuli instead of pain, simplifies verbal responses, removes “pain withdrawal” from motor responses, and distinguishes between normal and abnormal flexion. If an item could not be assessed, it was recorded as “Not testable” (NT). It is generally recommended that whole-body CT be performed as early as possible in patients with major trauma, so it may be useful to look for spinal injuries in this protocol [76,77,78,79,84,85].

#### 2.2.5. Transfer to a Final Care Facility

Despite previous attention to the issue of delays in transport to definitive care facilities, studies have shown that a significant proportion of patients with trauma still undergo tests such as CT performed prior to transport, contributing to an average delay of approximately 90 min [86]. Therefore, if imaging studies, such as CT, performed by the sending hospital do not lead to a change in the patient’s immediate course of treatment, expedited transfer to a definitive care facility should be considered before additional imaging studies are performed. The Situation, Background, Assessment, and Recommendation (SBAR) tool should be used to communicate information between healthcare providers for all patients, including the choice of appropriate mode of transport (helicopter or ground transport) and input of the care required on arrival at the final treatment facility [87].

## 3. Conclusions

Our trauma system continues to evolve, with positive results in reducing the number of preventable deaths. Evidence supporting this progress includes the growing implementation of prehospital interventions that target critical factors such as airway and breathing management, bleeding control, and rapid transport selection. For instance, the introduction of new protocols for airway management and bleeding control has been linked to improved survival rates in trauma patients. Studies show that the application of early hemorrhage control methods like tourniquets has drastically reduced mortality rates from traumatic bleeding. Similarly, the use of prehospital trauma life support (PHTLS) principles, including swift transport to trauma centers, has shown significant improvements in outcomes for patients with severe injuries. At the hospital level, the revised guidelines for advanced trauma life support (ATLS) have established a reliable framework for the initial assessment and management of trauma patients, which has helped ensure consistency in care and reduce errors during the early phases of trauma management. A study in a regional trauma center demonstrated that adherence to ATLS protocols led to a decrease in complications and better patient outcomes. These developments indicate not just a change in techniques or guidelines but a shift in the overall trauma management paradigm (Table 2). Although the global trauma system is changing rapidly, widespread adoption and use of prehospital trauma life support and advanced trauma life support are essential to further reduce preventable deaths due to trauma. These strategies are expected to improve the treatment outcomes of patients with trauma and ultimately reduce the preventive trauma death rate.

## Figures and Tables

**Table 1:** Updated classification of hemorrhagic shock.

Metrics	Class I	Class II (Mild)	Class III (Moderate)	Class IV (Severe)
Predicted blood loss	<15%	15~30%	31~40%	>40%
Heart rate	↔	↔/↑	↑	↑↑
Blood pressure	↔	↔	↔/↓	↓
Pulse pressure	↔	↓	↓	↓
Respiratory rate	↔	↔/↑	↔/↑	↑↑
Urine output	↔	↔	↔/↓	↓
GCS Score	Maintain normal	Maintain normal	Decrease	Significant reduction
Base deficit	0 to –2 mEq/L	–2 to –6 mEq/L	–6 to –10 mEq/L	–10 mEq/L or less
Need for blood products	Monitoring	May be required	Required	Massive transfusion

From American College of Surgeons. Advanced Trauma Life Support. 10th edition. Chicago: American College of Surgeons, Committee on Trauma; 2018 [61].

**Table 2:** Summarizing the articles discussed.

Components of Main Topic	
Prehospital	Airway management
	Chest decompression
	IV and IO
	Prehospital traumatic hemorrhage management strategies
	Spinal immobilization or spinal motion restriction
	Pelvic binder
	Treatment of traumatic brain injury
	Selecting a transport method
	Involvement of bystanders
	Traumatic cardiac arrest (TCA)
In-hospital	Airway
	Breathing
	Circulation
	Neurological assessment
	Transfer to a final care facility

## Data Availability

Not applicable.
